# Current status of chimeric antigen receptor engineered T cell-based and immune checkpoint blockade-based cancer immunotherapies

**DOI:** 10.1007/s00262-017-2007-x

**Published:** 2017-05-11

**Authors:** Upendra P. Hegde, Bijay Mukherji

**Affiliations:** University of Connecticut School of Medicine, 263 Farmington Avenue, Farmington, CT 06030 USA

**Keywords:** Tumor, Immunotherapy, CAR-T cells, ICI

## Abstract

Adoptive cell therapies with chimeric antigen receptor (CAR) engineered T cells (CAR-T) and immune checkpoint inhibition (ICI)-based cancer immunotherapies have lately shown remarkable success in certain tumor types. CAR-T cell-based therapies targeting CD19 can now induce durable remissions as well as prolong disease-free survival of patients with CD19 positive treatment refractory B cell malignancies and ICI-based therapies with humanized monoclonal antibodies against the T cell inhibitory receptors CTLA-4 and PD-1 as well as against the PD-1 ligand, PD-L1, can now achieve durable remissions as well as prolongation of life of a sizeable fraction of patients with melanoma and Hodgkin’s lymphoma and non-small cell cancers. Most importantly, these immuno-therapeutic treatment modalities have raised the possibility of achieving long-term “containment” as well as “cures” for certain types of cancer. While this represents major advances in cancer immunotherapy, both modalities come with considerable toxicities, including fatalities. Although more work will be needed to bring CAR-T cell-based therapies to the bedside for most major cancers and a good deal more will be needed to make ICI—alone or in combination with other treatment modalities—work more consistently and across most major cancers, these two treatment modalities stand out as superb examples of successful translation of bench research to the bedside as well as represent real progress in the field of cancer immunotherapy.

## Introduction

The field of cancer immunotherapy has gone through numerous peaks and valleys recording achievements and expectations as well as disappointments and disinterest. While monoclonal antibody-based therapies for cancer—in one iteration or another—has a fairly long history and has shown considerable success [1], BCG has been reasonably effective in superficial bladder cancer [2], and trials of tumor infiltrating lymphocytes (TIL) in melanoma have shown some success in a fraction of patients [3], the field of cancer immunotherapy has been waiting for the infusion of novel ideas and real success. Lately, a series of clinical trials with fully humanized monoclonal antibodies that are capable of blocking the inhibitory signaling pathways mediated through the inhibitory T cell surface receptors, CTLA-4 and PD-1—usually referred to as immune checkpoint inhibition (ICI)—and immunotherapy with a patient’s own T cells retooled to express a set of chimeric antigen receptor (CAR) exhibiting antibody specificities have shown durable complete remissions as well as prolongation of survival of patients with certain types of cancer [4, 5] and have emerged as remarkable success stories.

Both modalities have raised the tantalizing prospect for some cancer patients enjoying significant prolongation of life with their cancers “contained”—and if not “cured”—especially for advanced melanoma [4], treatment refractory acute lymphocytic leukemia [5], and for treatment refractory Hodgkin’s disease [6], and lately for non-small cell lung cancers [7]. While these results are impressive, both modalities come with substantial toxicities including fatalities at times. Fortunately, fatal side effects are relatively rare and most side effects have turned out to be “manageable”. Understandably, these new immune based approaches to cancer treatment have been viewed as breakthroughs in the field and both modalities have been extensively reviewed separately [4, 5, 8]. Given that these two new modalities of tumor immunotherapy work by harnessing the formidable power of T cells in immune responses and have led to an unprecedented excitement in the field, an unbiased review of these two, together, would be useful. Accordingly, our purpose here is to undertake a critical examination of the reason(s) for the excitement brought on by these two new cancer treatment modalities, especially considering our long and somewhat frustrating search for effective cancer immunotherapy.

## The principles underlying CAR-T cell-based ACT and ICI-based tumor immunotherapies

The therapeutic potential of adoptive transfer of a patient’s own T cells, activated and expanded ex vivo, in cancer immunotherapy is now well established [3]. The central idea behind making CAR-T cells for tumor immunotherapy is to harness the formidable power of T cells in anti-tumor responses by endowing them with the power to recognize relevant tumor-associated antigens through a set of receptors bearing the structure of an antibody, free from MHC restriction, and thus to enhance the scope of T cell-based adoptive immunotherapy. CAR-T cells therapy, therefore, represents ACT with T cells retooled to target a tumor-associated antigen through the precision and power of an antibody. Kuwana et al. [9] first described the concept of making chimeric T cell receptors. Shortly thereafter, Eshhar’s group [10] also published how to construct CAR-T cells. Given that a large number of such tumor reactive T cells bearing chimeric receptors could be generated from each patient, the translational potential of CAR-T cells in tumor immunothrerapy became obvious.

The fundamental principle behind ICI-based immunotherapy also aims to harness a robust T cell mediated immune response towards tumor-associated antigens by “taking the brake off” from the effector T cells. For, just as T cells are activated by signals through TCRs and co-stimulatory molecules, T cells also have receptors through which inhibitory signals can be transmitted—i.e., checkpoints or brakes. The operational principle underlying the ICI-based approach, therefore, is to unleash the host’s anti-tumor effector T cells by blocking these checkpoints with an antagonistic antibody—i.e., inducing a robust anti’-tumor T cell response by taking the “brake off”. Given that the checkpoint inhibition, on its own, does not provide TCR-based signals as well as co-stimulatory signals—two obligate necessities—for activating naïve T cells, it is believed that ICI primarily takes the “brake off” from antigen experienced T effector cells that have been rendered inactive, in vivo, by one mechanism or another as well as activates T memory cells. In this scenario, while it is widely believed that ICI does not prime naïve T cells, the precise mechanism(s) by which ICI works is not fully understood. Although it is fairly clear the major effect of the inhibitory reagents is on the effector T cells, given that regulatory T cells (Tregs) involved in the regulation of effector T cell activities also express inhibitory receptors, it has been suggested that anti-CTLA-4 antibodies work by potentiating T effector cells as well as by inhibiting Tregs [11]. This point; however, is yet to be fully settled.

## The development of CAR-T cell-based cancer immunotherapy

Soon after the methodology for engrafting a chimeric antigen receptor with antibody-like specificity into T cells became available, Rosenberg’s group at the NCI in collaboration with Eshhar at the Weizmann Institute demonstrated anti-tumor activities of adoptively transferred T cells made to express chimeric antibody/T cell receptors targeting folate receptors in a nude mouse model [12]. Thereafter, Rosenberg’s group translated the idea into a phase I clinical trial of CAR-T cells targeting folate receptors in ovarian cancer [13]. This trial did not lead to anti-tumor effectiveness, but showed that CAR-T cells can be relatively safely administered to human patients. Thereafter, in another early clinical trial of CAR-T cells engineered to express specificity for the cell adhesion molecule, L1 (CD171), in neuroblastoma, Park et al. [14] also could not demonstrate objective clinical responses, but observed that the adoptively transferred cells exhibited poor persistence. Shortly thereafter, Till et al. [15] from the City of Hope and the Fred Hutchinson Cancer Research Center also reported a proof-of-concept for tumor specific chimeric T cell receptor-based strategy in the treatment of non-Hodgkin’s lymphomas and Pule et al. [16] reported anti-tumor activities in neuroblastoma with CAR-T cell therapy targeting GD2 as a tumor-associated antigen.

Interest in tumor immunotherapy with CAR-T cells thereafter shifted to treating B cell malignancies targeting CD20/CD19. Using CD20/CD19 specific CAR-T cells, Jensen et al. [17] also found poor persistence of the CAR-T cells in patients due to anti-transgene rejection. Almost simultaneously, Kochenderfer et al. [18]; however, reported complete remission of CD19 positive advanced follicular lymphoma with the infusions of autologous CD19 CAR-T cells administered with IL-2. Interestingly, the treatment with CD19 CAR-T cells also led to the depletion of the patient’s B cell lineage from blood and bone marrow [18]. Soon, several groups confirmed these observations and reported that CAR-T cell therapy targeting CD19 could achieve complete remissions, including molecular remissions, in a fair fraction of CD19 positive leukemia that were refractory to traditional systemic treatments [19–23]. Most importantly, multicenter trials confirmed these basic observations and long-term follow-ups revealed that the complete remissions could be durable [24, 25] and raised the prospect of achieving “cures” of a sizeable fraction of refractory lymphocytic leukemia patients with CD19 CAR-T cell-based therapy [5, 26]. While this was clearly a positive development, CD19 CAR-T cell therapies came with substantial toxicities including fatalities (discussed later).

Understandably, the success with CD19 CAR-T cells in lymphocytic leukemia and lymphomas led to interests in extending this novel form of immunotherapy for other types of cancers targeting a variety of tumor-associated antigens. It also attracted substantial investments by the industries as CAR-T cells became viewed as “living drugs”. Unfortunately, despite considerable efforts by the academia and the industries, an equivalent(s) of CD19 for CAR-T cell therapy in tumors other than CD19 positive leukemias is yet to emerge. This is quite intriguing and it deserves some contemplation. CAR-T cell-based therapy is after all a form of immunotherapy and the issue of antigenic specificity has been a major pillar on which the effectiveness of any form of immune therapy rests. This raises the obvious question of what is so special about CD19? CD19 is not exactly a “leukemia specific” antigen, and CD19 is not expressed on lymphocytic leukemia cells all that differentially—qualitatively or quantitatively. Normal B cells express CD19; hence CD19 CAR-T cell therapy is associated with the loss of CD19 positive normal B cells from blood and bone marrow resulting in profound hypogammaglobulinemia [5, 18, 26]. Thus, the reason how CD19 has turned out to be a good target for CAR-T cells in lymphocytic leukemia and how other antigens and epitopes exhibiting similar expression pattern on other types of tumors including other hematological malignancies are not remains unclear!

### Design of CAR-T cells, dose schedule, additional measures, et cetera

A number of issues on CAR-T cell-based therapies remain unsettled. Among others, these include: (1) the ideal design of CAR-T cells (the inclusion of one or more than one co-stimulatory molecules and other molecules potentially affecting T cell function, survival, etc., viral vs. non-viral mechanism for gene transfer into T cells, ex vivo expansion methodology, et cetera); (2) the number of cells to be infused and the number of infusions for maximum benefit; (3) the need for and the nature of conditioning regimen (4), cytokine support to facilitate persistence of the infused cells, et cetera. While these issues are yet to be settled, most clinical trials have used the second generation CAR-T cells utilizing viral as well as non-viral gene transfer techniques, some form of pre-therapy lymphodepletion measure, exogenous IL-2 support or no cytokine support, and varied number of cells (10^5^–10^9^ cells per infusion) [5, 26]. Of note, CAR-T cell-based trials at multiple centers have tested infusions of a wide range of cell numbers and objective responses, including complete remissions, have been observed with different cell numbers [5, 26]. It is; however, by no means clear that large numbers of cells are needed to obtain complete remissions [5, 26]. In fact, complete remission has been achieved in chronic lymphocytic leukemia with as little as 1.4 × 10^5^ antigen specific population split into three consecutive days [19].

### Adverse events with CAR-T cell therapy and risk–benefit analysis

The side effects of CAR-T cell therapies can vary from mild constitutional syndrome (Flu-like symptoms, malaise, fever, etc.) to life-threatening situations requiring intensive care [5, 26–30]. The major toxicities usually result from: (a) inflammatory cytokines elaborated by the infused T cells from their cognate interactions with the target epitopes, (b) from secondary immune activation and from the activation of macrophages (usually referred to as “cytokine release syndrome or CRS” and macrophage activation syndrome), and (c) from massive target cell lysis (usually referred to as “tumor lysis syndrome”) resulting in high fever, hypotension, hypoxia, intravascular coagulation, etc. [5, 26–30]. The side effects can result from the CAR-T cells acting against epitopes “on target” as well as “off target”. At times, the toxicities can occur quickly, can lead to various organ (hepatic, gastrointestinal, respiratory, cardiovascular, endocrine, and neurological) dysfunctions, and at times they can be devastating resulting in death. Indeed, fatal pulmonary complication (such as seen with CAR-T targeting ERBB2 [31]), and a number of deaths from neurological complications—evidently from cerebral edema—lately encountered in the ongoing Juno Therapeutics trial. Although the reason for the fatal neurological side effects is yet to be fully clarified, the fatalities were attributed to Fludarabine used as a part of the preparative regimen.

Understandably, CAR-T cell therapy requires careful monitoring for side effects; and depending on the nature of the side effects, adverse reactions from CAR-T cell therapies call for quick supportive cares including intensive cares, as well as additional measures when needed. Additional measures for the major toxicities include the use of systemic steroids and the anti-IL-6 receptor blocking monoclonal antibody, tocilizumab, for CRS-related toxicities [25–28].

It should be pointed out that while the various serious adverse events following CAR-T cell therapy remain a matter of concern, most of the toxicities from CD19 CAR-T cell therapy are becoming “manageable” [5, 8, 26–30]. However, as CAR-T cell therapies designed to target other antigens in other tumor types enter into the clinics, they are likely to lead to different types of “on target and off target” adverse events. Further, as different pre-conditioning regimens get introduced, they can also lead to additional toxicities and can add to the side effects from the inflammatory responses by the infused T cells. Thus’, CAR-T cell therapy essentially navigates on uncharted territories. As such, a true evaluation of risk–benefit analysis of CAR-T cell therapy is presently not possible. Nonetheless, the benefits of durable complete remissions that a fair fraction of patients with B cell malignancies who have exhausted all standards of care can now enjoy with this form of therapy represent a remarkable development in cancer immunotherapy.

### The development of ICI-based cancer immunotherapies

In the early days of studies of signaling pathways in T cells, CTLA-4 was thought to be just another co-stimulatory receptor [32]. It was; however, soon learned that the ligation of CTLA-4 with an agonist, in fact, opposes CD28-driven co-stimulation and results in the down-regulation of T cell responses [33, 34]. As the role of CTLA-4 functioning as a brake in T cell responses became better understood, Allison recognized that taking the brake off from T cells might help unleash anti-tumor effector responses. Pursuing the idea in animal models, his group showed that the antibody mediated blockade of CTLA-4 indeed leads to tumor regression [35]. It took some time to take the idea to the bedside, but eventually the anti-CTLA-4 antibody was humanized, industries got involved, and humanized anti-CTLA-4 antibody was moved to clinical trials.

Several phase I–II trials quickly revealed anti-tumor activities of the inhibitory anti-CTLA-4 antibody [36–39] opening a novel strategy in cancer immunotherapy [40]. Interestingly, while several types of tumors showed positive response, the results of CTLA-4 blockade were most impressive in melanoma. Trials of anti-CTLA-4 blockade-based therapy continued and several multicenter-based trials of Ipilimumab, alone or in combination with gp100-based immunization, confirmed anti-tumor activities of the anti-CTLA-4 antibody Ipilimumab alone, and most importantly, showed its effectiveness in improving survival of melanoma patients [41].

Yet interestingly, the best overall response rates (95% CI) from the multicenter trials were not all that high (The response rates for Ipilimumab, gp100, and Ipilimumab + gp100 were 10.9, 1.5, and 5.7%, respectively). No complete response was noted with gp100 alone, and the CR rate for Ipilimumab and Ipilimumab + gp100 were 2 and 1%, respectively. However, the median survival rates were improved with Ipilimumab alone, gp100 alone, or with Ipilimumab + gp100 (The median survivals of the three groups were 10.1, 6.4 and 10 months, respectively). More importantly, the overall survivals of the three groups at 2 years (Kaplan–Meier survival curves) for the Ipilimumab, gp100, and Ipilimumab + gp100 groups were 23.5, 13.7 and 21.6%, respectively [41]. Further, the disease was stabilized in a significantly larger fraction of patients receiving Ipilimumab alone or receiving Ipilimumab + gp100 and objective responses in a fraction of patients receiving re-induction therapy either with Ipilimumab alone or with Ipilimumab + gp100, were noted. The response rates of the NCI trials were not substantially different. This trial showed that Ipilimumab alone can induce complete responses in a fraction of patients and that the combinations of Ipilimumab with IL-2 or with gp100 might be little bit better [42]. Understandably, the data from the multicenter trials showing improvement in response rate and improvement in overall survival with Ipilimumab [41] generated considerable excitement in the field.

Subsequently, additional trials were launched and outcome analyses of the initial trials continued. The analyses of ten subsequent prospective trials and two retrospective studies involving little over 1800 patients revealed overall survival rates of 24 and 26% at 3 years receiving Ipilimumab + gp100 or Ipilimumab alone with a fraction of patients living at the 10 years’ mark [43]. Similarly, a long-term follow-up of the NCI trial also revealed “durable” and “potentially curative tumor regressions” in melanoma with the combinations of Ipilimumab and IL-2 or Ipilimumab and gp100 (5-year survival rates of 25 and 23%, respectively, while the same with Ipilimumab alone being 13%) [43]. Of considerable interest, some patients in the NCI trial had shown continued tumor regressions over time eventually achieving complete tumor regressions even after stoppage of the treatment. This follow-up also showed that although relapses in some patients were noted after 3–4 years, the survival curve flattened at 3–4 years’ mark [43].

The development of ICI targeting PD-l and PD-L1 followed a similar path. Initially in a search for the genes involved in programmed cell death, Honjo’s group identified PD-1 as a programmed death associated molecule, hence the name programmed death for PD [44]. Thereafter Sharpe and Freeman, in collaboration with Honjo, discovered the natural ligands for PD-1—i.e., PD-L1 and PD-L2—and found that the engagement of PD-1 by the ligands negatively regulate T cell function pretty much the same way as CTLA-4 but, in a non-overlapping manner [45]. Around the same time, Chen’s group independently discovered PD-L1 that they called B7-H1 as a third co-stimulatory member of the B-7 family [46]. They went on to show that tumors cells often express the B7-H1/PD-1 ligand seemingly to escape host immune responses and recognized the potential for designing T cell-based cancer immunotherapy [47]. These collective observations led to the developments of reagents that could target PD-1 as well as PD-L1 as another approach to inhibitory receptor blockade-based tumor immunotherapy. In due time, phase I trials began with the anti-PD-1 antibody Nivolumab and the anti-PD-L1 antibody BMS-936559. Both trials included several different types of tumors and anti-tumor activities with both reagents were shortly reported [48, 49]. Another anti-PD-1 antibody, Pembrolizumab (initially called Lambrolizumab, developed by Merck) also entered clinical trial and showed essentially identical results [50].

Interestingly, the phase I trials with Nivolumab and Lambrolizumab and with the anti-PD-L1 antibody, BMS 936559, were not mature enough for median or overall survival data, the results of the first rounds of PD-1 as well as PD-L1 targeted trials nonetheless established a number of facts. First, it was quite clear that all three reagents have anti-tumor activities (the response rates against melanoma for Nivolumab, Lambrolizumab, and BMS 936559 were 28, 38, and 17%, respectively); second, Nivolumab and the anti-PD-L1 antibody exhibited varied degrees of clinical activities in tumors other than melanomas such as in lung cancer, renal cancer, and ovarian cancer); third, the reagents targeting anti-PD-1 were found to be effective in patients that have previously received Ipilimumab; and fourth, the toxicities of all three reagents seemed to be considerably less severe [48–50].

Subsequently, analyses of multiple phase III trials with anti-PD-1 antibodies involving much larger patient cohorts confirmed that not only both reagents showed anti-tumor activities, both anti-PD-1 antibodies prolonged median survivals (16.8 months for Nivolumab and 31 months for Pembrolizumab) as well as prolonged overall survival rates (43% for Nivolumab and 49% for Pembrolizumab at 2 years) [51, 52]. Importantly, in line with the Kaplan -Meier survival curve for melanoma patients receiving Ipilimumab flattening at the 3-year mark, the survival curves with the anti-PD-1 targeted therapies were also found to flatten at the 3-year mark [51, 52 **]**. Similarly, a recent extended follow-up of the Nivolumab trial [53], presented at the American Association of Cancer Research Annual Meeting 2016 at New Orleans, showed the overall survival curve plateauing at 4 years with 34% alive at 5 years.

A head to head comparison between the CTLA-4 blocking agent, Ipilimumab, and the PD-1 blocking agent, Pembrolizumab, has been carried out [54]. When the progression-free survival (PFS) and overall survival with the two were compared, the median estimates of PFS of patients receiving Pembrolizumab10 mg/kg every two weeks was found to be somewhat longer than that with Ipilimumab (5.5 vs. 2.8 months). The overall survival at 1 year was also significantly better with Pembrolizumab (74.1 vs. 58.2%, respectively). Similarly, the combination of Ipilimumab and Nivolumab has also been tested [55–57]. A phase I trial including nearly 100 patients receiving the two reagents revealed a response rate of about 40% with a 2-year survival of 79%. A subsequent randomized trial of the two compared with Ipilimumab alone showed a much better response rate with the combination (61% for the combination vs. 11% for Ipilimumab alone [57]. Finally, when a combination of Ipilimumab and the standard chemotherapeutic agent for melanoma, Dacarbazine, was compared with Dacarbazine and placebo alone, a significant improvement in overall survival of previously untreated melanoma patients receiving the combination was observed [58].

## ICI in cancers other than melanoma

ICI-based therapies for tumors other than melanoma have mostly been carried out with PD-1 blockade-based reagents. In fact, the very early PD-1 and PD-L1 blockade-based trials included renal cancers, non-small cell lung cancers, and ovarian cancers; and some measure of objective responses were observed in all of them [48, 49]. Shortly thereafter, objective responses with anti-PD-L1 reagent were also observed in bladder cancer [59]. However, the most impressive results with anti-PD-1/PD-L1 blockade-based treatments have been in refractory Hodgkin’s disease [6], in Merkel-cell carcinoma [60], and in non-small cell lung cancers [7]. Importantly, PD-L1 blockade-based treatment of PD-L1 positive non-small cell cancer—a tumor that had no effective treatment—has been quite remarkable. The progression-free as well as overall survivals in patients with this disease can now be extended with anti-PD-L1 reagents [7].

## Relation between response rates and survival from ICI

As pointed out earlier, the overall response rates and the complete response rates from ICI have been no better than 50 and 10%, respectively. Yet checkpoint blocking-based therapies have prolonged the overall survival of patients with melanoma. This seems to defy a cardinal point earlier established by cancer chemotherapeutic trials that showed that unless complete remissions were achieved in a large fraction of the patients, partial responses and “disease stabilization” do not improve overall survival. Thus, given that the complete remission rates in the ICI-based trials were no better than 10–15% and although some patients were likely to have undergone complete remission at a later date and some patients were likely to have received additional treatments for disease progression, the survival curve flattening at around 3–4 years is essentially unprecedented in the annals of treatment of metastatic melanomas, although the overall survival curve of patients treated with tumor infiltrating lymphocytes (TIL) has also been reported to flatten at around the same time [3].

## Adverse events with ICI and cost-benefit analysis

The adverse effects of ICI-based treatments include consequences of inducing T cell mediated immune responses, in vivo, somewhat polyclonally. Given that the reagents target a molecule that is expressed by most all activated T cells and as tumor tissues as well as normal tissues contain some activated T cells as well as given that T cells bearing specificities for a variety of normal tissue associated antigens exist, taking the “brake off” of all T cells leads to on target as well as off target toxicities. The toxicities mostly include inflammatory side effects resulting general debilities and effects from “inflammation” on a variety of organ systems such as the gut, skin, lung, liver, endocrine organs (such as pituitary) with occasional fatality [48–52, 63]. Of note, the incidence of adverse events and their severity with anti-PD-1-blocking agents are somewhat less than those with the CTLA-4 blocking agent [54–57]. The incidence and the severity of the adverse events with the combination of the two are; however, substantially higher than those with monotherapy [56]. Fortunately, most of these side effects are manageable. The management principles include, in addition to systemic supportive care, the use of immunosuppressive agent such as steroids, and in refractory cases, anti-TNF alpha agent, infliximab, anti-thymocyte globulin, and mycophenolic acid [56].

When the collective data on adverse effects of ICI-based treatments are taken into consideration, the toxicities of ICI remain substantial. Nonetheless, the composite data from clinical trials involving thousands of melanoma patients have clearly shown significant prolongation of progression-free median survivals as well as shown improvement in the overall survivals with a fraction of patients (around a third) remaining alive beyond 3 years. Significant benefits in patients with refractory Hodgkin’s disease, advanced Merkel-cell carcinoma, and in non-small cell lung cancers have also been achieved. Thus, it would be fair to conclude that the benefits clearly outweigh risks and that ICI-based cancer immunotherapies do represent a true advancement in the field.

## Factors affecting clinical responses

A great deal of effort has been mounted to figure out factors and biomarkers that would be predictive of treatment response and better outcome. Among the various factors that have been considered, given that tumor cells often express PD-L1or L2, the ligands for PD-1, the expression of PD-L1 or L2 was correlated with response and this was thought to be a predictive factor [61]. Subsequent analyses; however, failed to establish a clear relationship between PD-L1 expression by the tumors and treatment outcome. In this context, it should be pointed out that PD-1 blockade has been found to be remarkably effective in treatment of refractory Hodgkin’s disease [6] and in non-small cell lung cancers [7]. Given that Reed-Sternberg cells in Hodgkin’s disease often exhibit amplification of chromosome, 9p24.1 that codes for the PD-1 ligands [62] the possibility exists that in certain tumors showing amplification of 9p24.1, PD-L1 expression might be a factor for good outcome.

The other potentially useful biomarker for response was thought to be the intensity of immune activities in the tumors, in situ. More CD8+ T cell-based immune infiltrates and the pattern of infiltration within the tumor tissues—serving as footprints of host immune activities and making a tumor “hot”—better is the chance of response [63, 64]. In addition, it has been suggested that PD-1 blockade induces responses by inhibiting adaptive resistance [64]. While, the evidence of brisk infiltration of CD8+ T cells, in general, the evidence of preferential accumulation of clonal T cells, in particular, and inhibition of “adaptive resistance” have been associated with good response, a definitive relationship between the intensity and the nature of the immune infiltrate and inhibition of adaptive resistance and treatment outcome is yet to be clearly established.

Another potentially useful biomarker of positive result that has gained considerable attention is the mutation load (“genetic basis”) in a given tumor [65]. It has been suggested that tumors that exhibit more mutations are likely to attract more host immune response—especially to such mutated “neo-antigens”—and thus likely to respond to ICI-based treatments [66]. Again, while this has been forwarded as another determinant of positive outcome, more studies will be needed to establish it as a truly positive biomarker.

Finally, and somewhat ironically, the extent of autoimmune adverse effects was initially thought to be a marker for better response to the earlier CTLA-4 blockade-based trials [67]. Subsequent trials; however, have not validated this finding. Thus, despite substantial efforts at defining useful biomarkers for response to ICI-based treatments, it would be fair to say that nothing has so far been proven truly useful.

## Discussion

CAR-T cell- and ICI-based tumor immunotherapies have changed the very tenor of the discussions (claims of advances and admissions of failures) on the “value” of cancer immunotherapy. An accomplished tumor immunologist who had “kept an open eye” and felt “years of frustrations” in the field recently told the authors of this article that “it has been a revolution”. Setting this and other personal views (unpublishable personal communications) as well as the institutional approbation [68] aside, presently it will not be inappropriate to say that these two forms of tumor immunotherapies have finally proved that immune-based cancer therapies can be made to work in humans—albeit in certain tumor types and at some cost. Unfortunately, while the adverse effects can be serious—including fatality in some—the side effects mostly represent reflections of unleashing robust immune responses at the tumor sites as well as at certain types of normal tissues. Fortunately, while the adverse events can be quite serious and unpredictable, most side effects are becoming manageable.

CD19-CAR-T cell therapy has been granted orphan status by the FDA. Intense efforts are underway to extend both CAR-T cell-as well as ICI-based treatments across other tumor types and to improve outcomes when employed alone or in combination with other agents. FDA has already approved the use of combined CTLA-4 and PD-1 blockade-based therapy. Additional combination strategies such as ICI with GM-CSF, with GM-CSF producing cell-based vaccine, and with other stimulatory signals such as with 4-1BB or OX40, IDO inhibitors or combination of ACT and ICI are under consideration. A variety of other combinations with immune inhibition [such as with LAG-3, TIM-3, BTLA, etc.], chemotherapeutic agents and irradiation—as additive or designed to make a tumor more “antigenic”, etc., are in the drawing board. Additionally, the combination of ICI with neo-epitope-based immunization to make the treatment more personalized is also actively pursued. Time will tell which of these strategies are likely to improve results. In this context and encouragingly, the effectiveness of the combination of ACT with IL-21-primed polyclonal cytolytic T cells and anti-CTLA-4 antibody in a melanoma patient that had failed prior treatments with ACT and anti-CTLA-4 has recently been reported [69]. Indeed, if the history behind the development of effective combination chemotherapies for cancer is any guide, the expectation for positive outcomes from combination immunotherapy for cancer would not be inappropriate.

This brings us to the interesting findings of long survival of patients who do not achieve complete remissions from anti-CTLA-4 as well as anti-PD-1 blockade-based therapies. Traditionally, the operative goal of any systemic anti-cancer therapy has been sustained complete remissions in a sizeable fraction so as to improve overall survival and to achieve “cure”. Interestingly, given that ICI achieves complete remissions in no more than 15% of the patients yet the survival curves of patients receiving CTLA-4-as well as PD-1 blockade-based therapies flatten after 3 to 4 years for a sizeable fraction (a respectable third) of the patients, it is tempting to argue that we are seeing something unique that can be best described as “the containment of cancer”. This seems to defy the lesson learned from earlier efforts at finding “curative” chemotherapy and it is somewhat analogous to what has been achieved with HIV–AIDS [70] and with chronic myeloid leukemia [71] as patients affected by these two diseases enjoy long life with treatments that does neither achieve total neutralization of the offending virions nor completely eliminate the leukemic cells. If the data with ICI-based therapies hold, this will be not only quite impressive; this could represent a new legitimate goal in the management of cancer—i.e., keeping cancers from progressing! The results of ICI-based cancer immunotherapy seem to suggest that this goal is achievable.

Meanwhile as innovative clinical trials continue, factors influencing outcome—i.e., response vs. no-response to therapies—get further analyzed, and relevant scientific issues behind CAR-T cells and ICI-based strategies undergo careful examination, we believe that it can now be said with a definite measure of confidence that the evidence for genuine progress in the field of cancer immunotherapy with CAR-T cell- and ICI-based strategies has been compelling; and that there are reasons to expect further progress with these two basic approaches in cancer immunotherapy.

## Abbreviations

4-1BB: TNF receptor superfamily 9
ACT: Adoptive cell therapy
BTLA: B-and T lymphocyte attenuator
CAR-T cell: Chimeric antigen receptor engineered T cell
CR: Complete response
CRS: Cytokine release syndrome
CTLA-4: Cytolytic T lymphocyte antigen-4
ERBB2: Epidermal growth factor receptor 2
ICI: Immune checkpoint inhibition
IDO: Indoleamine 2,3-dioxygenase
LAG-3: Lymphocyte-activation gene 3
NCI: National Cancer Institute
OX40: TNF receptor superfamily 4
PD: Programmed death
PD-L: Programmed death ligand
PFS: Progression-free survival
TCR: T cell receptor
TIM-3: T cell immunoglobulin and mucin-domain containing-3
Tregs: Regulatory T cells
